# Analysis of flow-induced transcriptional response and cell alignment of different sources of endothelial cells used in vascular tissue engineering

**DOI:** 10.1038/s41598-023-41247-6

**Published:** 2023-09-01

**Authors:** Diana M. Rojas-González, Aaron Babendreyer, Andreas Ludwig, Petra Mela

**Affiliations:** 1 Department of Biohybrid & Medical Textiles (BioTex) at Center of Biohybrid Medical Systems (CBMS), AME-Institute of Applied Medical Engineering, Helmholtz Institute, RWTH Aachen University, Forckenbeckstr. 55, 52074 Aachen, Germany; 2 Chair of Medical Materials and Implants, Department of Mechanical Engineering, School of Engineering and Design and Munich Institute of Biomedical Engineering, Technical University of Munich, Boltzmannstr 15, 85748 Garching, Germany; 3 Institute of Molecular Pharmacology, Medical Faculty, RWTH Aachen University, Pauwelsstr. 30, 52074 Aachen, Germany

**Keywords:** Tissue engineering, Gene expression analysis

## Abstract

Endothelialization of tissue-engineered vascular grafts has proven crucial for implant functionality and thus clinical outcome, however, the choice of endothelial cells (ECs) is often driven by availability rather than by the type of vessel to be replaced. In this work we studied the response to flow of different human ECs with the aim of examining whether their response in vitro is dictated by their original in vivo conditions. Arterial, venous, and microvascular ECs were cultured under shear stress (SS) of 0, 0.3, 3, 1, 10, and 30 dyne/cm^2^ for 24 h. Regulation of flow-induced marker KLF2 was similar across the different ECs. Upregulation of anti-thrombotic markers, TM and TPA, was mainly seen at higher SS. Cell elongation and alignment was observed for the different ECs at 10 and 30 dyne/cm^2^ while at lower SS cells maintained a random orientation. Downregulation of pro-inflammatory factors SELE, IL8, and VCAM1 and up-regulation of anti-oxidant markers NQO1 and HO1 was present even at SS for which cell alignment was not observed. Our results evidenced similarities in the response to flow among the different ECs, suggesting that the maintenance of the resting state in vitro is not dictated by the SS typical of the tissue of origin and that absence of flow-induced cell orientation does not necessarily correlate with a pro-inflammatory state of the ECs. These results support the use of ECs from easily accessible sources for in vitro vascular tissue engineering independently from the target vessel.

## Introduction

The performance of clinically available synthetic conduits for small vessel replacement is hampered by thrombus formation due to insufficient hemocompatiblity of the materials used^1,2^. Early efforts to address this problem focused on recreating the functionality of the native endothelium by lining the luminal surface of the grafts with a monolayer of endothelial cells (ECs). To date, this is still a viable approach for the fabrication of tissue-engineered vascular grafts (TEVG)^3–5^. Different ECs sources have been used for this purpose, common sources include human umbilical vein ECs (HUVEC)^6–8^, ECs from tissue microvasculature^9,10^, endothelial progenitor cells^11^, and stem cells^12^.

In the human body, ECs play a key role in maintaining vascular homeostasis in response to mechanical and chemical signals, and are involve in providing an anti-thrombotic surface and modulating immune responses^13,14^. ECs are directly exposed to shear stress (SS), which regulates their function by activation of intracellular signal pathways and transcription factors, as well as by modulating gene expression^15,16^. Exposure to physiological laminar SS leads to a resting state of the endothelium characterized by an anti-thrombotic, anti-inflammatory, and anti-proliferative ECs phenotype^17^; it also induces morphological changes in the ECs and protects them from oxidative stress^17^. Conversely, ECs exposure to disturbed flow induces a state of the cells where pro-inflammatory and pro-coagulant pathways are activated^15,18^, which has been shown to promote the development and progression of vascular diseases such as atherosclerosis^19–23^. Reports in literature have also described that activated ECs fail to orient in flow direction implying an existing link between ECs alignment and their altered state^24–28^. In fact, cell elongation and alignment in flow direction has been adopted as a characteristic trait of ECs and is often used as an evaluation criterion for ECs functionality in vitro^29,30^.

Understanding the behavior and changes of ECs is crucial for the progress of research in vascular tissue engineering. This not only applies to TEVG fabrication, but also other treatment alternatives (e.g., endothelialized stents), and to the development of in vitro models that can recapitulate physiological and pathological conditions to study vascular disease progression and thus serve as platform for development of devices and therapeutic options. Several in vitro studies have investigated the effect of different flow patterns^31–33^ on ECs or have focused on the identification of pathways or molecules influenced by SS^34–36^. Such studies usually use ECs from one vascular bed^8,37,38^ or ECs from animal sources^39–41^.

In vascular tissue engineering, the selection of ECs for research purposes is often influenced by cell availability, accessibility of the cell source, and ease of isolation rather than the type of vessel to be replaced^12^. HUVEC represent one of the most common cell types used for in vitro ECs research. In fact, an analysis on publications between 2013 and 2018 found that 59% of the studies use HUVEC as a source of human ECs^42^. Results of such studies are often assumed to be transferrable to other EC types^43^. Nevertheless, in vivo*,* ECs located in different parts of the vascular tree are considered to have morphological, physiological, and phenotypical differences^44^. For instance, arterial ECs form tighter intercellular junctions and are usually thicker, longer, and narrower than venous ECs^43,45,46^. Furthermore, at gene level, specific arterial or venous markers have been identified, such as EphrinB2 for arterial ECs and EphB4 for venous ECs^43^. Variations among ECs, particularly their different morphologies, have also been linked to the different SS magnitudes across the vascular tree^43^. Arterial magnitudes of SS are considered to be between 10 and 70 dyne/cm^2^, while venous SS magnitudes go from 1 to 5 dyne/cm^2^^18,46–48^. In the case of the microvasculature a wide range of SS from 1 to 95 dyne/cm^2^ has been described^29,49^.

Considering the heterogeneity of the human endothelium, the question arises as to whether the choice of ECs for in vitro research should be instead driven by the type of ECs of the target application. In this work we set out to evaluate the morphological and phenotypical changes of human ECs derived from different vascular beds (umbilical cord arteries and vein, and pulmonary and adipose microvasculature) when subjected to a range of laminar SS. The aim of our study was to analyze the behavior of the different ECs and establish whether their performance in vitro is dictated by their original in vivo environment. To this end, the cells were exposed to SS in the range from 0.3 to 30 dyne/cm^2^, which included magnitudes considered physiological as well as non-physiological for the different cell sources. The evaluation consisted of the analysis of cell orientation and the detection of a wide panel of genes related to inflammation, thrombogenicity, oxidative stress, and cell physiology aiming to have a complete overview on the state of the cells and to provide valuable insight into the in vitro behavior of different types of ECs of human origin under flow.

## Results

### Gene expression profiles

**Figure 1:**
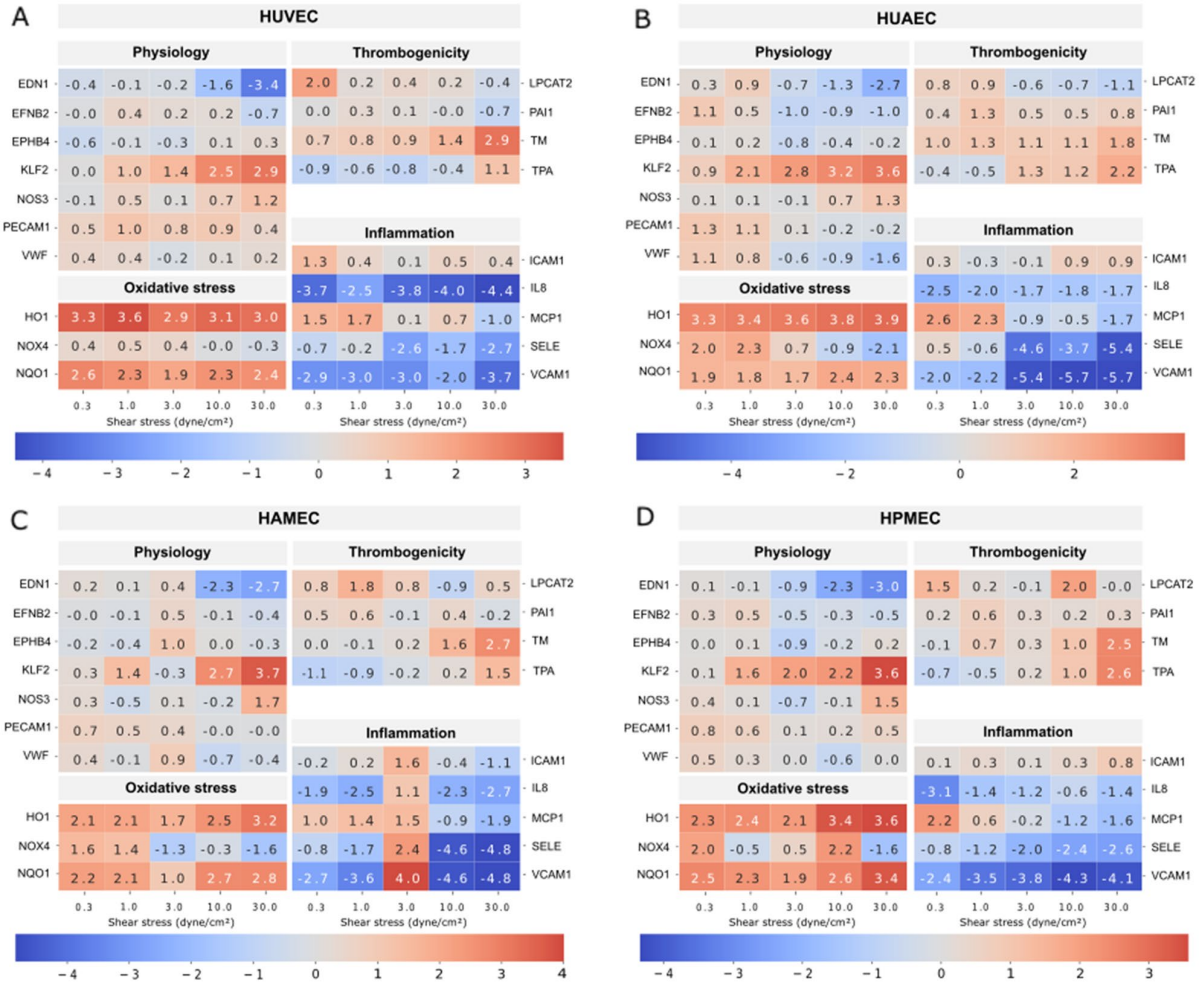
Heat maps depicting fold changes for all measured markers in HUVEC (**A**), HUAEC (**B**), HAMEC (**C**), and HPMEC (**D**). The numbers represent the fold change in log2 scale of the relative mRNA expression levels of the samples normalized to their corresponding static controls. Heatmaps were created using the seaborn package in python (v3.11, https://seaborn.pydata.org/index.html).

**Figure 2:**
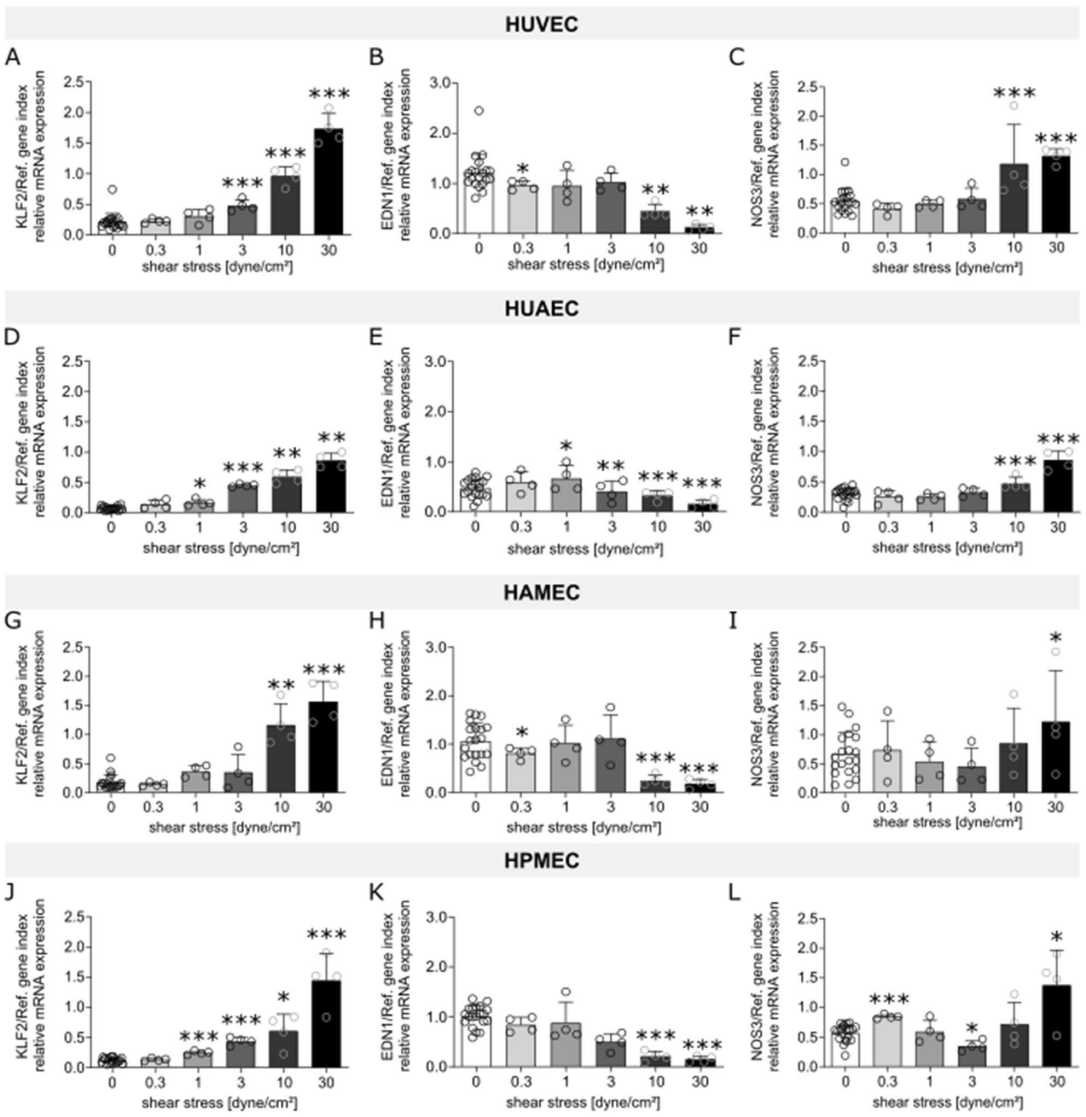
mRNA expression of markers related to endothelial cell physiology (KLF2, EDN1, NOS3) for HUVEC (**A**–**C**), HUAEC (**D**–**F**), HAMEC (**G**–**I**), and HPMEC (**J**–**L**) at the different magnitudes of shear stress. Statistically significant differences to the static control are indicated by asterisks (*p < 0.05, **p < 0.01 and ***p < 0.001).

**Figure 3:**
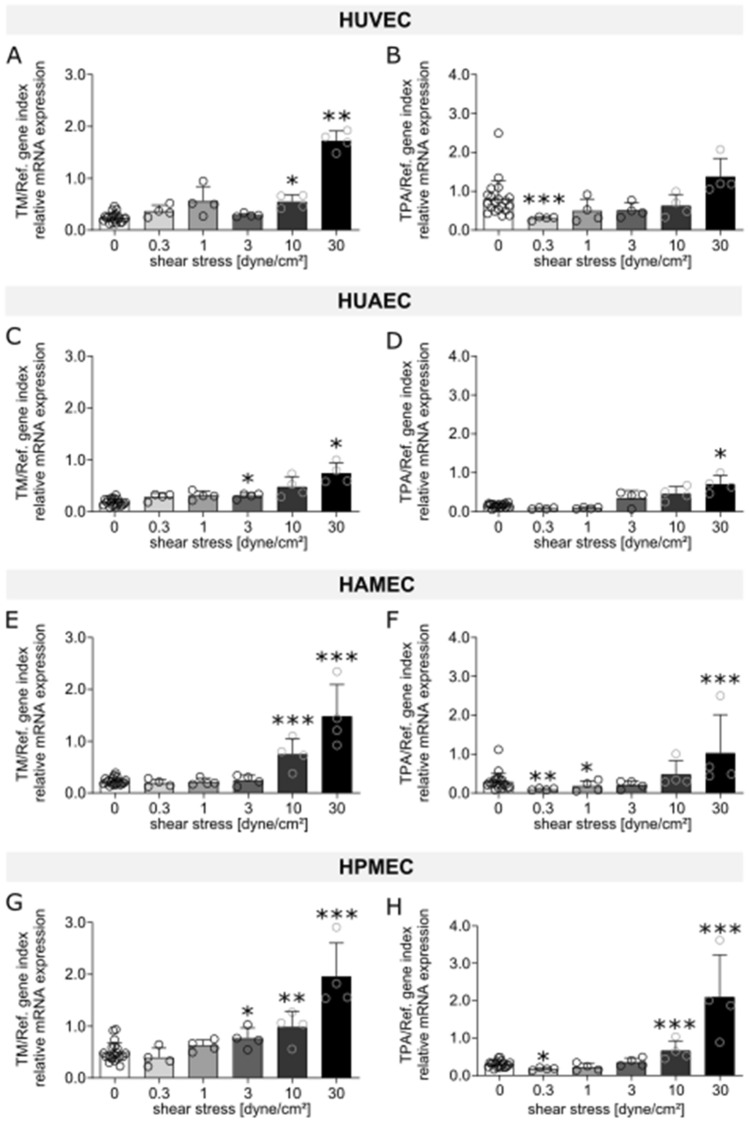
mRNA expression of markers related to thrombogenicity (TM, TPA) for HUVEC (**A**,**B**), HUAEC (**C**,**D**), HAMEC (**E**,**F**), and HPMEC (**G**,**H**) at the different magnitudes of shear stress. Statistically significant differences to the static control are indicated by asterisks (*p < 0.05, **p < 0.01 and ***p < 0.001).

**Figure 4:**
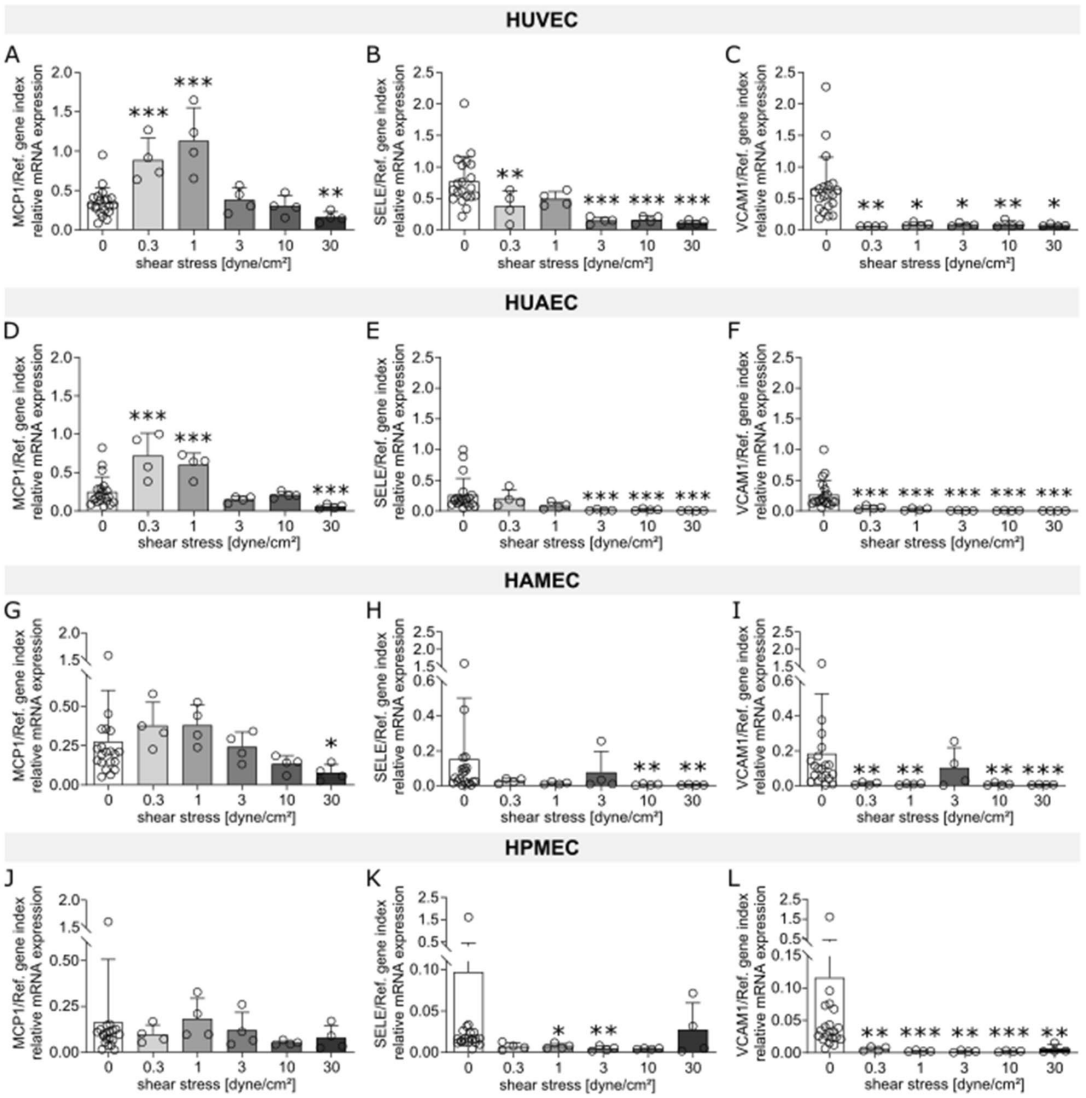
mRNA expression of markers related to inflammation (MCP1, SELE, VCAM1) for HUVEC (**A**–**C**), HUAEC (**D**–**F**), HAMEC (**G**–**I**), and HPMEC (**J**–**L**) at the different magnitudes of shear stress. Statistically significant differences to the static control are indicated by asterisks (*p < 0.05, **p < 0.01 and ***p < 0.001).

**Figure 5:**
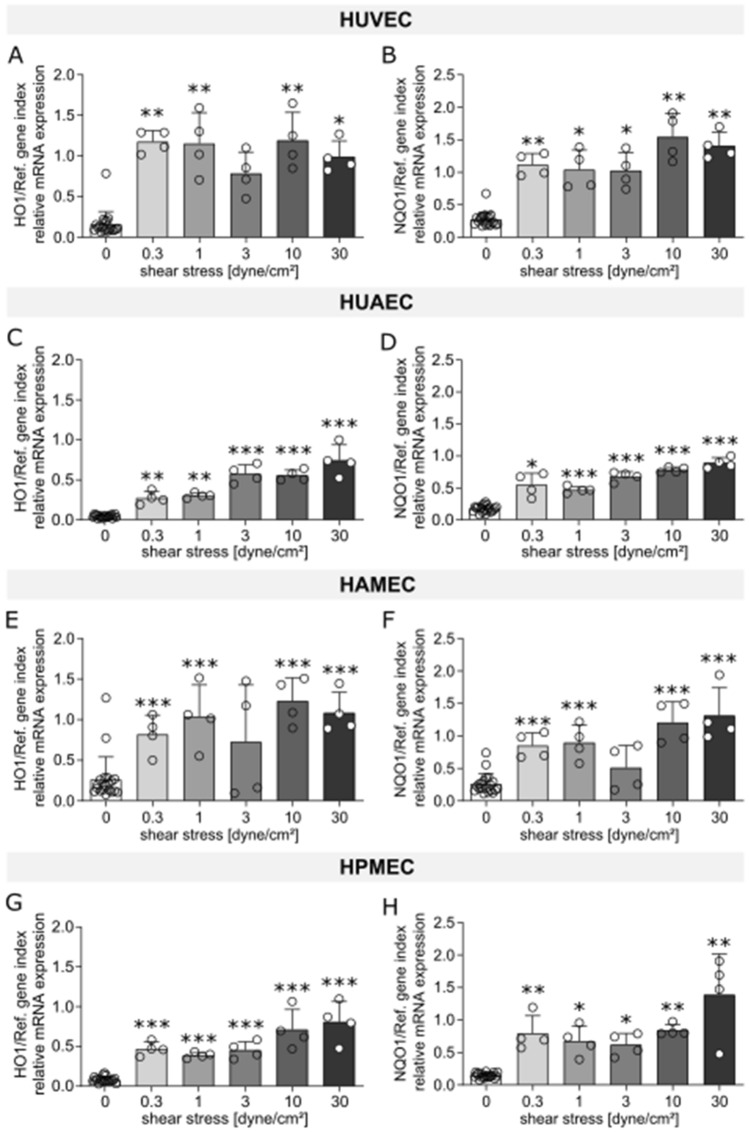
mRNA expression of markers related to oxidative stress (HO1, NQO1) for HUVEC (**A**,**B**), HUAEC (**C**,**D**), HAMEC (**E**,**F**), and HPMEC (**G**,**H**) at the different magnitudes of shear stress. Statistically significant differences to the static control are indicated by asterisks (*p < 0.05, **p < 0.01 and ***p < 0.001).

The effect of flow exposure on the different ECs was evaluated through the analysis of mRNA expression levels of different markers, which were chosen based on reports found in literature on genes that are influenced by shear stress and that can be linked to the functional aspects of vascular physiology, thrombogenicity, oxidative stress, and inflammation. Heat maps for human umbilical vein ECs (HUVEC; Fig. 1A), human umbilical artery ECs (HUAEC; Fig. 1B), human adipose derived microvascular ECs (HAMEC; Fig. 1C), and human pulmonary microvascular ECs (HPMEC; Fig. 1D) show the fold change of the different dynamic samples compared to their respective static controls for all the markers measured (Table 1). Here, we could identify a group of 10 markers in which mRNA up- or down-regulation due to exposure to SS was observed across the different ECs. Such group is comprised of genes of well-known flow-regulated proteins: KLF2, EDN1, and NOS3 (Fig. 2); genes of pro-inflammatory molecules: MCP1, SELE, and VCAM1 (Fig. 3); genes of key anti-thrombotic proteins: TM and TPA (Fig. 4); and genes of major oxidative stress-related molecules: HO1 and NQO1 (Fig. 5). Figures for the mRNA expression of the additional markers listed in Table 1 are found in the supplementary material for HUVEC (Suppl. Fig. S1), HUAEC (Suppl. Fig. S2), HAMEC (Suppl. Fig. S3), and HPMEC (Suppl. Fig. S4).

**Table 1:** Panel of markers evaluated through qPCR.

Category	Name	Abbreviation	Source
EC physiology	Krüppel-like factor 2	KLF2	^50,51^
	Endothelin 1	EDN1	^13,16^
	Nitric oxide synthase 3	NOS3	^13,15,79^
	von Willebrand Factor	vWF	^13,79^
	Platelet endothelial cell adhesion molecule	PECAM1	^13,15,79^
	Ephrin-B2	EFNB2	^79^
	Ephrin type-B receptor 4	EPHB4	^79^
Thrombogenicity	Thrombomodulin	TM	^13,16,50^
	Tissue plasminogen activator	TPA	^13,64^
	Lysophosphatidylcholine acyltransferase 2	LPCAT2	^80^
	Plasminogen activator inhibitor	PAI1	^17,50,79^
Inflammation	Interleukin 8	IL8	^50,79^
	Vascular endothelial cell adhesion molecule	VCAM-1	^16,50,51^
	Endothelial leukocyte adhesion molecule	SELE	^17,50,56^
	Intercellular adhesion molecule	ICAM-1	^16,50,58^
	Monocyte chemoattractant protein	MCP-1	^16,50^
Oxidative stress	Heme oxygenase	HO1	^50,62^
	NAD(P)H dehydrogenase	NQO1	^50,62^
	NADPH oxidase 4	NOX4	^17^

#### EC physiology

A trait of ECs is the flow-mediated induction of the transcription factor KLF2, which is responsible for the regulation of the majority of SS-sensitive genes^50^. A shear-dependent regulation of this marker was observed for the different ECs analyzed. A significant increase in mRNA expression of KLF2 was detected starting from 1 dyne/cm^2^ for HUAEC (Fig. 2D) and HPMEC (Fig. 2J), from 3 dyne/cm^2^ for HUVEC (Fig. 2A), and from 10 dyne/cm^2^ for HAMEC (Fig. 2G). Expression of genes encoding for EDN1 and NOS3, both targets of KLF2, was also measured. In the case of EDN1, a marker related to vasoconstriction, significant downregulation was observed at the highest SS magnitudes applied, 10 and 30 dyne/cm^2^, for all cells. Nevertheless, for HUAECs this downregulation was already seen starting from 3 dyne/cm^2^ (Fig. 2B,E,H,K). Furthermore, mRNA expression of NOS3, critical effector molecule for vasodilation, showed significant upregulation at 10 and 30 dyne/cm^2^ for HUVEC (Fig. 2C) and HUAEC (Fig. 2F) while for the microvascular cells this significant increase was seen at 30 dyne/cm^2^ and additionally at 0.3 dyne/cm^2^ for HPMEC (Fig. 4I,L).

Additional markers such as PECAM1 and vWF, which are typically expressed by ECs, were also evaluated. In general, for PECAM1 no relation between the extent of mRNA expression and the magnitude of SS was recognized. For HUVEC, PECAM1 was slightly upregulated at all SS magnitudes (Suppl. Fig. S1A), while in their arterial counterpart this was only observed at low SS, namely 0.3 and 1 dyne/cm^2^ (Suppl. Fig. S2A). For the microvascular cells, HPMEC had a similar behavior to the arterial cells (Suppl. Fig. S4A), and in HAMEC this upregulation was additionally seen at 3 dyne/cm^2^ (Suppl. Fig. S3A). For vWF, also no relation between the mRNA expression and the SS magnitude was observed for the different ECs except for HUAEC (Suppl. Figs. S1B–S4B). This is better noticed in the heat maps, where fold change values for vWF stayed in general close to the static control for HUVEC (Fig. 1A), HAMEC (Fig. 1C), and HPMEC (Fig. 1D), while for HUAEC a decrease in fold change with increasing SS was observed (Fig. 1B). Furthermore, mRNA expression of genes encoding for EFNB2 and EPHB4, which have been reported to be selectively produced in arterial and venous ECs phenotypes respectively, did not show a preferential up- or down-regulation in any of the cell types analyzed (Suppl. Figs. S1C,D–S4C,D).

#### Thrombogenicity

Expression levels of anti-thrombotic markers TM and TPA were analyzed for the different cell types. For TM, mRNA expression showed upregulation at the two highest SS magnitudes, with a significant increase for all ECs at 30 dyne/cm^2^ (Fig. 3A,C,E,G). For the lower SS, 0.3 to 3 dyne/cm^2^, increased expression was observed for the venous (Fig. 3A) and arterial (Fig. 3C) ECs. For HAMEC, mRNA levels at these low SS remained close to the static control (Fig. 3E), while for HPMEC, an overall increase in mRNA expression with increasing SS magnitude was detected (Fig. 3G). In the case of TPA, regulation patterns for the different ECs had varied behaviors, however a common point among all ECs was a visible upregulation at 30 dyne/cm^2^ and downregulation at 0.3 and 1 dyne/cm^2^ (Fig. 3B,D,F,H). Overall, upregulation of anti-thrombotic markers was achieved under exposure at high magnitudes of shear stress, in particular at 30 dyne/cm^2^ where the increased expression of both TM and TPA reached the highest expression levels for all types of ECs (Fig. 3).

Additional markers included pro-thrombotic markers LPCAT2 and PAI1. Expression of LPCAT2 showed an increase at SS of 0.3 and 1 dyne/cm^2^ followed by decreased expression levels at the following SS magnitudes in both HUVEC (Suppl. Fig. S1E) and HUAEC (Suppl. Fig. S2E). For PAI1, HUVEC showed mRNA levels close to the control for all SS except 30 dyne/cm^2^ where a significant downregulation was detected (Suppl. Fig. S1F). On the other hand, HUAEC exhibited a significant mRNA increase for all SS magnitudes, however not in a SS-dependent manner (Suppl. Fig. S2F). In microvascular cells mRNA levels stayed in general close to the static control for both LPCAT2 and PAI1 (Suppl. Figs. S3E,F, S4E,F).

#### Inflammation

A potential inflammatory phenotype was studied with the mRNA expression level of the chemokine MCP-1 and the adhesion molecules E-selectin and VCAM1. The mRNA expression of MCP-1 was reduced with higher magnitudes of SS (10 and 30 dyne/cm^2^) across the different ECs types (Fig. 4A,D,G,J). At the two lowest SS magnitudes, significant upregulation was detected for HUVEC (Fig. 4A) and HUAEC (Fig. 4D). Microvascular cells showed also a tendency for upregulation at these low SS, which for HAMEC included also the 3 dyne/cm^2^ magnitude (Fig. 4G–J). This effect is better recognized in the heat maps for both HAMEC (Fig. 1C) and HPMEC (Fig. 1D). For the genes of the adhesion molecules SELE and VCAM1, a strong effect of flow was observed*.* The mRNA expression of both markers was preferentially downregulated in all the different ECs after flow exposure, regardless of the SS level (Fig. 4). For HAMEC at 3 dyne/cm^2^ a higher level of expression was observed compared to the other SS evaluated (Fig. 4H,I), however this is mainly caused by an outlier in the data, the effect of which is also visible in the fold change for the different inflammatory markers in the HAMEC heatmap (Fig. 1C). Expression of IL8 was also marked by preferential downregulation of this marker at the different SS magnitudes for all ECs (Suppl. Figs. S1H–S4H). Furthermore, mRNA levels of ICAM-1, another adhesion molecule usually expressed in ECs, exhibited no influence of the SS magnitudes in its expression pattern (Suppl. Figs. S1G–S4G). In summary, flow exposure shows a general anti-inflammatory effect in all ECs types at the different SS magnitudes with regards to the expression of markers related to adhesion molecules and chemotactic factors involved in the recruitment of immune cells. This effect is even stronger at higher SS where it is accompanied by downregulation of the chemoattractant protein MCP-1.

#### Oxidative stress

NQO1 and HO1 play a key role in preventing production of reactive oxygen species (ROS). Both markers showed upregulation in all cells regardless of the vascular bed of origin and the level of SS to which they were exposed (Fig. 5). This result points to a protective effect against oxidative stress of flow on ECs. Additional downregulation of mRNA expression of NOX4, a marker related to ROS production, was detected at 30 dyne/cm^2^ for the different ECs (Suppl. Figs. S1I–S4I). This effect is better observed in the heat maps for each of the cells (Fig. 1). Furthermore, upregulation of NOX4 was seen at the two lowest SS magnitudes for all cells, except HPMEC where this is true at 0.3 dyne/cm^2^.

### Gene expression comparison across ECs types

**Figure 6:**
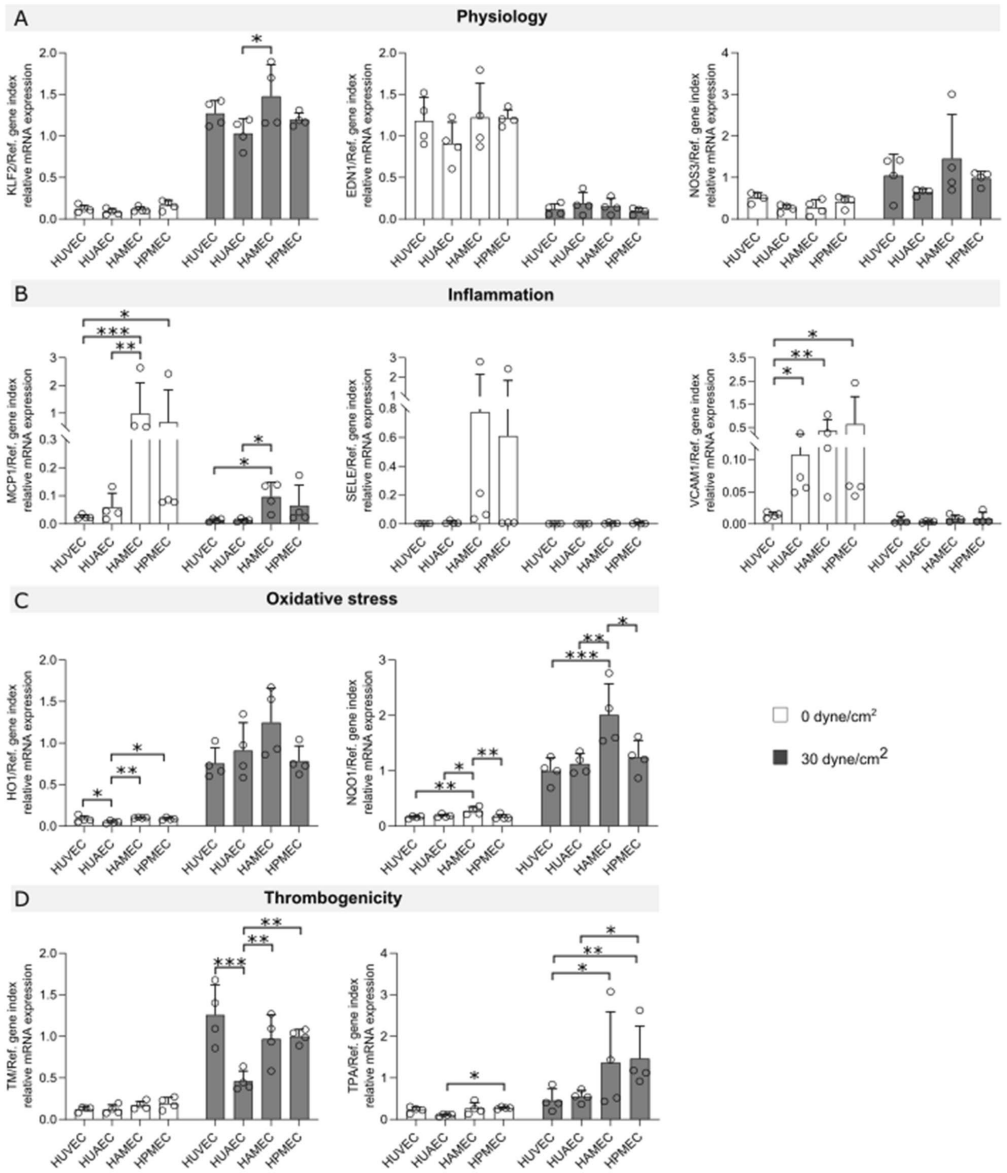
Comparison of mRNA expression levels across endothelial cells from different vascular beds exposed to 0 and 30 dyne/cm^2^. Statistically significant differences across different ECs types are indicated by asterisks (*p < 0.05, **p < 0.01 and ***p < 0.001).

mRNA expression levels were directly compared across the various ECs to eventually highlight differences arising from their vascular origin. For this comparison, samples from cells exposed to 30 dyne/cm^2^ were used because, as shown before, significant regulation of mRNA expression was seen at this SS magnitude for the panel of 10 markers shown in previous figures. Overall, trends in up- or down-regulation of mRNA expression remained the same for the various ECs (Fig. 6). Of interest is the expression at static conditions of the inflammatory markers MCP-1, SELE, and VCAM1, for which microvascular cells showed higher expression (Fig. 6B). Nevertheless, after flow exposure, the different cell types reached similar levels of downregulation. Similarly, for ROS-protective markers HO1 and NQO1, differences at 0 dyne/cm^2^ were observed, however exposure to 30 dyne/cm^2^ lead to increased mRNA levels for all the ECs. HAMEC showed a stronger upregulation in particular for NQO1 (Fig. 6C). In the group of markers related to thrombogenicity, increased expression was observed for all cells after flow exposure. Microvascular cells showed differences in their mRNA expression compared to the arterial and venous ECs for TPA (Fig. 6D). For TM, microvascular and venous ECs reached a similar expression level, with differences to HUAEC that had the lowest upregulation level of the group. Taken together, our results show that regardless of the vascular origin and differences in expression levels at static conditions, high shear stress drives the marker expression in the same way reaching a similar end-point across cell types that is an anti-inflammatory, anti-thrombotic, and ROS protective ECs phenotype.

### Principal component analysis (PCA)

**Figure 7:**
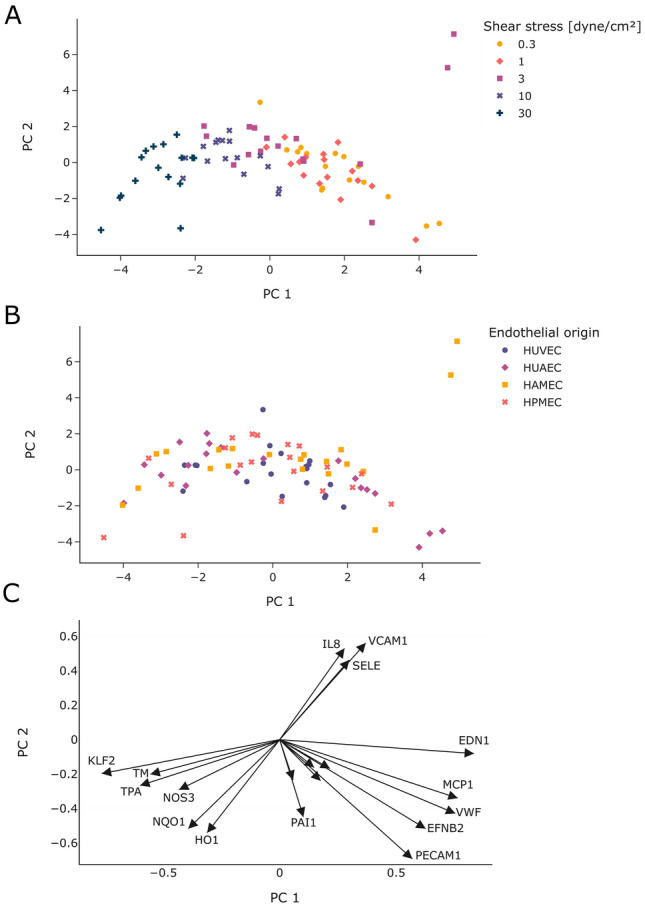
Principal component analysis of mRNA expression data. The results are presented as scattered plots where each symbol represents one sample. Samples were colored for the different shear stress levels (**A**) or the different endothelial cell type (**B**). The loadings for the different genes were plotted as arrows (**C**).

In addition to the classical evaluation of the qPCR experiments, a multidimensional PCA was performed. The PCA showed a good separation of cells conditioned with high SS (10 and 30 dyne/cm^2^) from those cultured with lower magnitudes of SS (Fig. 7A). In contrast, no pattern was observed with respect to the different origins of the endothelial cells (Fig. 7B). Similar to that, there was also no pattern observed for the different coatings used during cell expansion (Suppl. Fig. S5). Considering the loadings for the different genes, two groups of genes can be identified that are important in differentiating cells cultured under high or low SS (Fig. 7C). While the mRNA expression of KLF2, TM, TPA, NOS3, NQO1 and HO1 have a greater impact on cells cultured under high SS, the mRNA expression of EDN1, MCP1, VWF, EFNB2 and PECAM1 is important when conditioning cells with the lower magnitudes of SS used in the experiments. Noticeably, two samples of HAMEC at 3 dyne/cm^2^ were clearly distinguishable from the other samples (Fig. 7A,B), which was determined by the mRNA expression of the pro-inflammatory genes IL8, VCAM1 and SELE. The other markers examined in this work showed a rather minor influence and were therefore not included in the graph.

### Endothelial cell alignment

**Figure 8:**
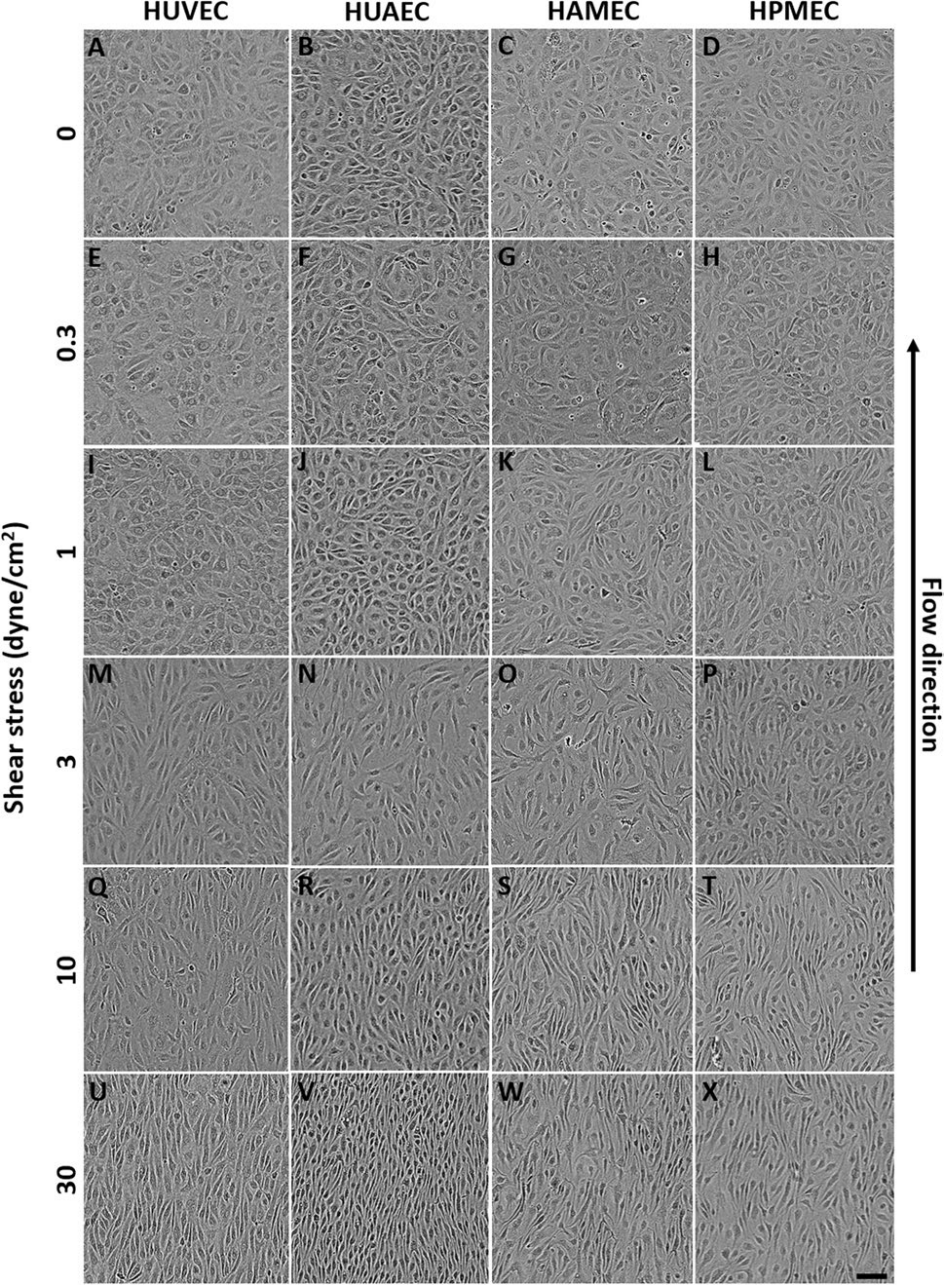
Phase contrast images of the different types of endothelial cells after 24 h of static culture (**A**–**D**) and flow exposure at shear stress magnitudes of 0.3 (**E**–**H**), 1 (**I**–**L**), 3 (**M**–**P**), 10 (**Q**–**T**), and 30 dyne/cm^2^ (**U**–**X**). Scale bar: 100 µm.

**Figure 9:**
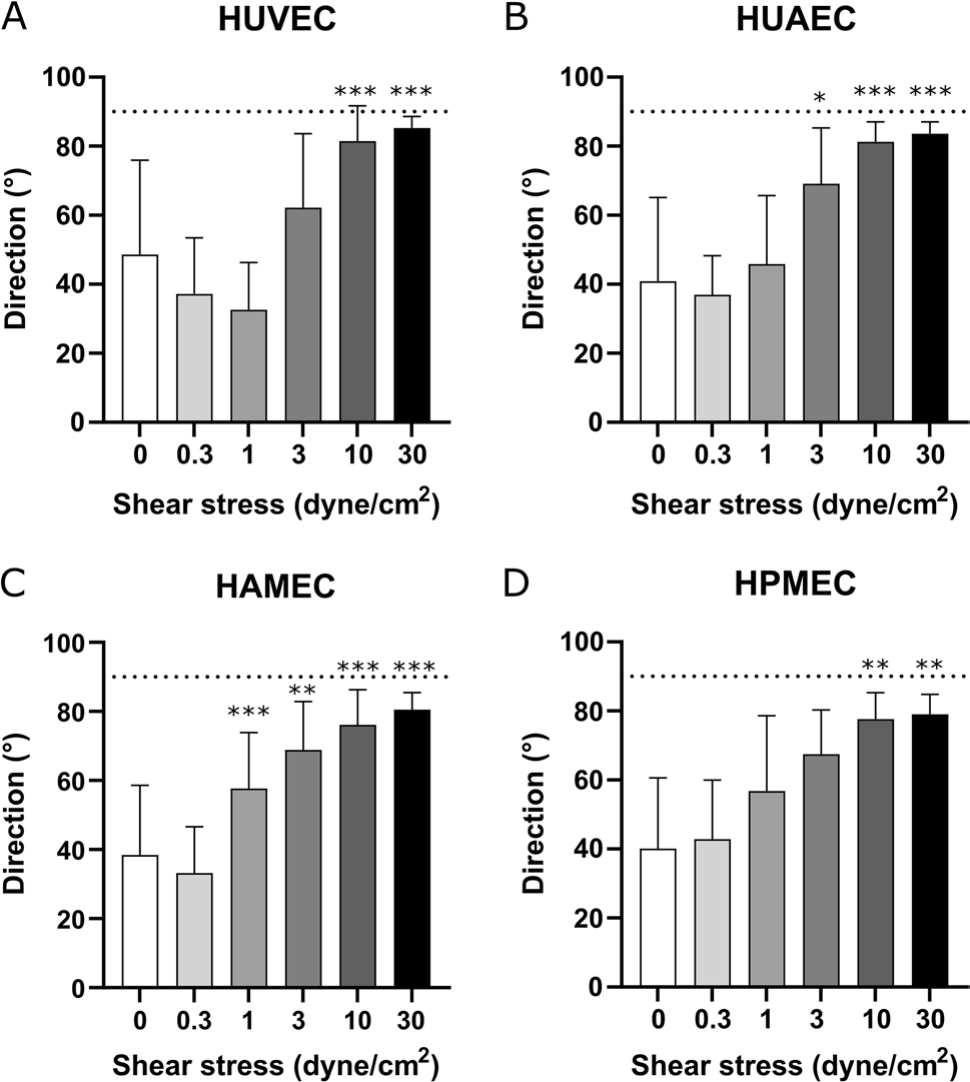
Analysis on preferred cell direction at the different shear stress magnitudes for HUVEC (**A**), HUAEC (**B**), HAMEC (**C**), and HPMEC (**D**). Statistically significant differences to the static control are indicated by asterisks (*p < 0.05, **p < 0.01 and ***p < 0.001). Dotted line indicates 90°.

After 24 h of exposure to flow, changes in cell morphology and orientation were observed for the different ECs types. At 0.3 dyne/cm^2^, all ECs appeared with a cobble-stone morphology similar to the static control (Fig. 8A–H). This was also the case for arterial and venous cells at 1 dyne/cm^2^ (Fig. 8I,J), while for the microvascular cells already some elongated cells were observed (Fig. 8K,L). At 3 dyne/cm^2^ the different cells appeared more elongated although orientation was still mostly random (Fig. 8M–P). Quantitative image analysis of HUVEC and HUAEC supported these observations (Fig. 9A,B). At 0.3 and 1 dyne/cm^2^ mean direction angles remained below 60° and standard deviations were high similar to the static controls (Fig. 9A,B). At 3 dyne/cm^2^ the overall mean direction of the cells increased compared to the lower SS magnitudes, nevertheless the standard deviations remained high, corresponding to the onset in cell elongation without orientation in flow direction observed in the brightfield images. For the microvascular cells, this increase in mean direction angle was detected earlier, at 1 dyne/cm^2^, and progressively increased with the increasing SS (Fig. 9C,D). Cell elongation and orientation along flow direction was visible in all ECs types when conditioned at a SS of 10 and 30 dyne/cm^2^ (Fig. 8Q–X). At these SS magnitudes, mean direction angles were closer to 90° (flow direction) and their values showed statistically significant difference compared to the static control in all the cells (Fig. 9). In summary, the different types of ECs responded similarly to flow exposure reaching comparable orientations at high SS. Nevertheless, microvascular cells appeared to be more sensitive exhibiting orientation changes with a lower stimulation compared to the ECs from the larger umbilical vessels.

## Discussion

In this work we present a comprehensive analysis of the effect of flow exposure on gene expression and cell orientation of different types of human-derived ECs relevant for basic research, not only in vascular tissue engineering but in endothelial cell research in general. Despite a few minor differences, the gene expression profile of the ECs analyzed showed consistent and broad similarities among the various types of vascular beds, which was also well reflected in the results of the PCA. Starting with flow-induced upregulation of KLF2 in a SS-dependent manner, which was observed for all types of ECs. This goes in line with reports in literature for this transcription factor^50,51^. This result also validates the presence of an undisturbed flow in our experiments, since, as demonstrated by Wang et al., in ECs under disturbed flow conditions KLF2 expression is suppressed^52^. Furthermore, laminar SS has been reported to play an important part in maintaining vascular homeostasis and retaining a vasoprotective ECs state through the regulation of this transcriptional factor^13,50^, which has also been described to act as a switch between an atheroprone and atheroresistant EC phenotype^53^.

KLF2 has a role in maintaining an anti-thrombotic and anti-inflammatory state on the ECs, which is of great importance for TEVG as the main causes for graft failure are thrombosis and intimal hyperplasia, processes where the expression of pro-inflammatory molecules plays a crucial role^54,55^. Leukocyte adhesion to the endothelium is one of the initial events of atherogenesis, which is usually mediated by adhesion molecules such as VCAM-1, SELE, and ICAM-1^56^. In fact, in the work by Chappel et al., a pro-inflammatory state in HUVECs was defined by increased surface expression of those molecules^57^. Contrary to this, in our study, significant downregulation of VCAM-1 and SELE was observed for all ECs at the different magnitudes of laminar SS, while expression levels of ICAM-1 stayed close to the static control and only in some instances a slight upregulation was observed. Together, these results indicate that all different ECs acquire a similar anti-inflammatory phenotype under laminar flow.

In addition, literature reports that ICAM-1 can be constitutively expressed in ECs and regulated by shear stress to some extent, and that increased levels of this adhesion molecules is observed in pro-inflammatory processes such as atherosclerosis^58^. Besides no clear increase in ICAM-1 expression, our results also showed no shear dependent regulation of this marker. Contrary results were reported by Nagel et al.^59^, where flow exposure of HUVEC for 48 h to a range of laminar SS between 2.5 and 46 dyne/cm^2^ resulted in upregulation of ICAM-1 linked to increase adhesion of lymphocytes. The authors observed that this selective upregulation was time-dependent and force-independent, as ICAM-1 increased progressively during the duration of the flow exposure and its amount was not related to the level of SS applied. The time frame chosen by the authors was 48 h, however at 24 h, a time frame similar to ours, ICAM-1 expression was already clearly increased, which did not happen in our case. The duration chosen for our study is a time frame that is often used in ECs research as these cells respond almost instantly to flow exposure^36^. Studies have shown that changes in cell morphology and alignment, as well as regulation of flow-responsive genes happen in this time frame^37,51^.

Vascular protective effects of KLF2 are also exerted through the induced expression of NOS3. This marker not only acts as a vasodilator trough nitric oxide (NO) production, it also decreases platelet adhesion and aggregation, has anti-inflammatory properties, and acts as a free radical scavenger^60^. While our cells did not show significant regulation of NOS3 at low SS, at high SS (10 and 30 dyne/cm^2^), where also the highest KLF2 expression was found, upregulation of NOS3 was observed. Zhang et al.^61^, reported a similar finding on HUVEC with the difference that in their study low SS (4.2 dyne/cm^2^) downregulated NOS3 expression, however an explanation to this behavior was not provided by the authors. Genes HO1 and NQO1, which play a role in preventing ROS production and therefore protecting the cells from oxidative stress, were upregulated for all cell types at all points even when alignment was not present, indicating a protective state of our ECs against oxidative stress. Redox imbalance has been reported to cause endothelium dysfunction, a precursor of cardiovascular diseases such as atherosclerosis^62^.

Shear-induced KLF2 has an anti-thrombotic effect and regulates the expression of key genes that confer the desired anti-thrombotic properties of the ECs^17,53^. Our results showed increased expression of anti-thrombotic markers TM and TPA, particularly at high SS where also the highest KLF2 expression was found, while expression levels of pro-thrombotic marker PAI1 stayed close to the static control. Lin et al.^63^, showed that overexpression of KLF2 in HUVEC induced TM and reduced PAI1. Our results for TM are consistent with this, however PAI-1 regulation was not observed. Our results are consisted with those reported for HUVEC by Diamond et al.^64^, where PAI1 remained unchanged when exposed to arterial magnitudes of SS (15 and 25 dyne/cm^2^), while preferential regulation of anti-thrombotic marker TPA took place.

In terms of morphological changes, cell elongation and alignment in flow direction has been considered a typical response of ECs to SS both in vivo and in vitro^65^. Due to the diverse hemodynamic conditions along the vascular tree, we hypothesized that such changes in cell morphology would be evident in vitro for each ECs type at SS magnitudes that are present at their vascular bed of origin. Nevertheless, our data showed that the different ECs responded quite similarly to the flow exposure. This differs from a previous report by Baeyens et al*.*, where exposure of HUVEC and lymphatic ECs to a range of SS of 2–60 dyne/cm^2^ for 16 h, resulted in cell alignment at different SS levels for the different ECs^66^. Lymphatic cells showed alignment at 4–6 dyne/cm^2^ while HUVEC aligned at 8–20 dyne/cm^2^. Our results for HUVEC are similar to these findings. The authors linked these differences in the set point for alignment to differences in the levels of VEGFR3 in each of the ECs types. This however was not measured in our work.

In this work, for ECs derived from the umbilical blood vessels, an onset of cell elongation was observed from 3 dyne/cm^2^ and orientation in flow direction was evident at 10 and 30 dyne/cm^2^. Saw et al., reported a SS of 28.1 ± 7.3 dyne/cm^2^ for the umbilical artery and of 5.2 ± 1.6 dyne/cm^2^ for the umbilical vein^67^. In this regard, the SS level at which cell orientation occurred for both HUVEC and HUAEC in our study (10 dyne/cm^2^), would already fall out of the physiological SS range of their blood vessel of origin. This would suggest that the SS from their original vascular bed does not influence the in vitro response of these ECs types. This was further supported by the downregulation of certain pro-inflammatory genes, upregulation of anti-oxidant genes, and induction of KLF2 expression, all pointing to a resting state of these cells from applied SS magnitudes of 0.3 dyne/cm^2^ and higher. For the microvasculature, SS magnitudes starting from 1 dyne/cm^2^ are reported as physiological^29,49^. In our in vitro study, HAMEC and HPMEC showed morphological changes at 1 dyne/cm^2^ and 3 dyne/cm^2^ although orientation in flow direction was only achieved at higher SS. A similar result was reported by Ischibazawa*, *et al.^68^, who exposed human retinal microvascular ECs to laminar SS in a range of 1.5 to 100 dyne/cm^2^. The microvascular cells exhibited cell alignment at SS ≥ 6 dyne/cm^2^.

In summary, a non-activated ECs state would be here characterized by upregulation of anti-thrombotic and redox-protective markers accompanied by downregulation of those considered as pro-thrombotic, pro-inflammatory, and pro-oxidative. Our findings in gene expression profiles show that the different ECs were in a non-inflammatory and non-oxidative state in the whole range of SS. The panel of genes described in relation to thrombosis did not exhibit an expression pattern that lead to a clear determination of the pro- or anti-thrombotic state of the cells. However, due to the preferential upregulation of TM and lack of regulation of PAI1 it could be said that cells are more prone to be anti-thrombotic. Taken together, our results on cell alignment and gene expression suggest that failure to align under flow does not indicate an activated state of the ECs. Furthermore, as cell elongation and alignment were seen in all cells at the same SS level, it suggests that a specific SS magnitude might be needed in order to see such changes, but this might not necessarily depend on the physiological conditions of the vascular bed of origin. Reports in literature have also pointed out to differences in EC response depending on substrate hardness and different flow profiles^52,69,70^. In this regard it should be kept in mind that this work was carried out with ECs cultured on a hard substrate and exposed to laminar unidirectional flow. It could be that the substrate stiffness and the type of flow are critical parameters driving the phenotype of ECs under culture, therefore examining the differences caused by these factors is part of our future work.

Overall, the similarity in the response of the different ECs types points to a reset in the cell memory which could be caused by the plasticity characteristic attributed to this cell type that has been reported in literature^71,72^. Previous work by Aranguren et al., indicated differences in marker expression between freshly isolated and cultured ECs, which further points to such reset^73^. Our findings provide a comprehensive gene expression profile of different types of ECs cultured under physiologically relevant SS magnitudes. The fact that the different ECs analyzed showed a similar response to laminar SS could bring some flexibility when choosing a source for ECs for in vitro studies. It could also endorse the use of ECs that do not match the target application for the fabrication of endothelialized TEVG. However, the presented mRNA expression data needs to be further supported with functional analysis (e.g., platelet adhesion experiments, measurement of NO production, analysis of protein regulation, and chemokine release) in future experiments. If these experiments will confirm the present results, this could bring the use of cell-based TEVG closer to clinical translation as autologous and easily accessible sources, such as microvascular ECs from fat tissue biopsies, could be safely used.

## Materials and methods

### Cell culture

HAMEC and HPMEC were purchased from ScienceCell (Carlsbad, USA); and Promocell (Heidelberg, Germany), respectively; HUAEC and HUVEC were isolated from umbilical cords provided by the Department of Gynecology at the University Hospital Aachen in accordance with the human subjects’ approval of the ethics committee of the Medical Faculty of RWTH Aachen University, Germany (EK 2067; EK241/18). Tissues were collected only after informed consent was obtained from patients or legal guardians. All methods involving human materials were carried out in accordance with the relevant guidelines and regulations, including the Declarations of Helsinki, as well as good laboratory and scientific practice. The isolation protocol followed is described elsewhere^74,75^. Briefly, umbilical cords were washed with phosphate buffered saline (PBS; Gibco) and then 1 mg/mL collagenase (Sigma-Aldrich) was injected into the lumen of the vessels for enzymatic removal of the cells. The isolated ECs were seeded on pre-coated (Gelatin or Collagen IV) tissue culture flasks (Greiner Bio-One) and further incubated to allow for cell expansion. HPMEC as well as two donors from HUAEC and HUVEC were cultured on flasks coated with collagen IV (Sigma), while HAMEC and the rest of the HUAEC and HUVEC donors were cultured on flasks coated with gelatin (Sigma). In order to ensure a better comparison, all ECs were cultured using the same endothelial growth medium (EGM-MV2; Promocell) and used between passages 2 and 4 for the experiments.

### Flow experiments

On the day of the experiment, cells were detached from culture flasks using accutase (Sigma), resuspended in EGM-MV2 medium, and seeded on non-coated ibidi µ-slides (Ibidi, Germany) at a density of 40.000 cells/cm^2^. After an incubation time of 2.5 h to allow for cell attachment, the slides were connected to the Ibidi pump system to generate unidirectional steady flow for 24 h. Depending on the geometry of the µ-slide used, the flow rate was adjusted as specified by the manufacturer to yield the desired SS. For experiments carried out with SS of 0.3, 1 and 3 dyne/cm^2^ slides with a channel height of 0.8 mm were used, whereas for experiments at 10 and 30 dyne/cm^2^ a channel height of 0.2 mm was chosen. ECs seeded in 24-well plates without coating and using the same seeding density as for the µ-slides were used as static controls. Each experiment with either HUAEC or HUVEC was performed with cells isolated from four different donors, and each experiment with either HAMEC or HPMEC was performed with cells from two different donors.

### Real time quantitative PCR (qPCR)

qPCR was used to determine the mRNA expression levels of different ECs markers related to inflammation, thrombogenicity, oxidative stress, and ECs physiology as listed in Table 1. Cellular mRNA was obtained using the RNeasy Mini Kit (Qiagen, Germany) with the addition of a homogenization step using the QIAshredder columns (Qiagen, Germany), and an on-column genomic DNA removal step as recommended in the protocol from the manufacturer. The RNA extracted was quantified using a spectrophotometer (NanoDrop, Thermofisher). For each set of experiments equal amounts of RNA were reverse transcribed using QuantiTec reverse transcription kit (Qiagen, Germany) and PCR reactions were performed using TB Green® Premix Ex Taq™ II SYBR green (Takara Bio Inc., Japan) according to the manufacturer’s instructions. The specific primers and annealing temperatures are listed in Supplementary Table S1. All PCR reactions were run on a CFX Connect Real-Time PCR Detection System (Bio-Rad) with the following protocol: 40 cycles of 10 s denaturation at 95 °C, followed by 10 s annealing at the indicated temperature and 15 s amplification at 72 °C. The mRNA levels of the genes of interest were normalized to the mRNA level of different reference genes. The most stable reference genes were determined with the geNorm algorithm included in the qbase + software (biogazelle, Belgium). Based on these results, glyceraldehyde 3-phosphate dehydrogenase (GAPDH) and TATA-binding protein (TBP) were chosen as reference genes. Since normalization was always performed against these two reference genes, we used the term reference gene index (ref. index) to make the y-scale clearer when presenting the data. PCR efficiency was determined from the uncorrected RFU values using LinRegPCR version 2020.0^76^. Relative quantification was performed with the CFX Maestro Software 1.1 (Bio-Rad). Additionally, heat maps were produced using the seaborn package in python and were used to present the fold change of the mRNA levels of the dynamic samples against their respective static controls.

### Alignment analysis

Images of the cells on the µ-slides were taken before and after flow exposure using a phase contrast microscope (IncuCyte® ZOOM, Essen Bioscience) at four points along the axis of the channel. For the static controls, images were taken at nine different points equally distributed throughout the well in a 3 × 3 grid. All images were taken with a 10 × magnification and had a region of interest of 2.15 mm^2^. Cell orientation was analyzed using the directionality plugin for Fiji, an open source image processing application based on Image J (NIH, USA)^77^. For the analysis, three images of each experiment were included and an area of 1 mm^2^ was considered per image. An angle of 90° was defined as parallel to the flow direction and 0° as perpendicular to it. The plugin provided a frequency distribution showing the number of cells oriented at the different angles. In images where a peak in the distribution was detected a Gaussian function was fitted by the program and information on the preferred direction of the cells (center of the Gaussian) was obtained. For a clearer presentation of the data, when a preferred direction angle was > 90° the correspondent supplementary angle was used instead. Figures were produced using GraphPad Prism (GraphPad Software).

### Principal component analysis (PCA)

PCA of the qPCR data was performed in python using the Scikit-learn module^78^. Prior to the PCA, the data was standardized using the StandardScaler of Scikit-learn. For the graphical representation of the PCA results, Plotly Express (Plotly Technologies Inc., Canada) was used. The samples were colored depending on the shear stress level, the endothelial cell type or the coating during cell propagation. The loadings for the different genes were plotted as arrows, whereas the length of the arrow represents the value of the loading.

### Statistical analysis

Quantitative data is shown as mean ± standard deviation (SD). The statistical analysis was conducted using the general mixed model analysis (PROC GLIMMIX, SAS 9.4, SAS Institute Inc.). Data was analyzed for the optimal distribution using Akaike Information Criterion (AIC), the Bayesian Information Criterion (BIC), residual plots, and the Shapiro–Wilk test as diagnostics. If necessary, the donor was set as a random term to assess for donor-specific differences. In the case of heteroscedasticity, according to the covtest statement, the degrees of freedom were adjusted by the Kenward-Roger approximation. If the data still did not represent a normal distribution (according to the Shapiro–Wilk test), a non-parametric Kruskal–Wallis test was performed (GraphPad Prism, GraphPad Software). Multiple comparisons were corrected by false discovery rate (FDR). A p-value < 0.05 was considered significant.

### Supplementary Information

Supplementary Information.

## Data Availability

The data generated during and/or analyzed in the current study are available from the corresponding authors upon reasonable request.
