# Supplementary material for: Analysis of flow-induced transcriptional response and cell alignment of different sources of endothelial cells used in vascular tissue engineering
Source: Sci Rep. 2023 Sep 1;13:14384. doi: 10.1038/s41598-023-41247-6 (PMC10474151; doi:10.1038/s41598-023-41247-6)
Supplement: Supplementary file 1 — Supplementary Information. [file 41598_2023_41247_MOESM1_ESM.pdf]

Supplementary data

Analysis of flow-induced transcriptional response and cell alignment of different sources of endothelial cells used in vascular tissue engineering

Diana M. Rojas-González[1,2,a], Aaron Babendreyer[3,a]*, Andreas Ludwig[3,b], Petra Mela[1,2,b]*

[1] Department of Biohybrid & Medical Textiles (BioTex) at Center of Biohybrid Medical Systems (CBMS), AME-Institute of Applied Medical Engineering, Helmholtz Institute, RWTH Aachen University, Forckenbeckstr. 55, 52074 Aachen, Germany.

[2] Chair of Medical Materials and Implants, Department of Mechanical Engineering, School of Engineering and Design and Munich Institute of Biomedical Engineering, Technical University of Munich, Boltzmannstr 15, 85748 Garching, Germany.

[3] Institute of Molecular Pharmacology, Medical Faculty, RWTH Aachen University, Pauwelsstr. 30, 52074 Aachen, Germany.

[a, b] Equal contribution

Corresponding authors:
Petra Mela: petra.mela@tum.de
Aaron Babendreyer: ababendreyer@ukaachen.de

Supplementary table S1. Primer sequences and annealing temperatures used for RT-qPCR

Gene name	Primer sequences		Annealing temperature
	Forward	Reverse	
KLF2	AAA GAC CAC GAT CCT CCT	CTT ATT TCT CAC AAG GCA TCA C	59 °C
EDN1	TGA AGC CAT AGC CTC CAC	GAA GAG ACC AAA GCA GTT ACC AC	60 °C
NOS3	CGA GTG AAG GCG ACA ATC CT	GCT GCA AAG CTC TCT CCA TTC	60 °C
VWF	TAC CAC AAC CAC CTG CCT	GTA AGT GAA GCC CGA CCG A	57 °C
PECAM1	CAG CCA ACT TCA CCA TCC	GAG AGC ATT TCA CAT ACG AC	57 °C
THBD (TM)	AGA GAA GAG ACA AAC ACC T	TCC ACA AGA CCA GTA GAG	57 °C
PLAT (TPA)	TGC TAC TTT GGG AAT GGG	GTT CTG TGC TGT GTA AAC CT	57 °C
LPCAT2	GTA GAA GTT GAG TTT ATG CCA G	CTT ACG AAC ACC ATC CCA	58 °C
SERPINE1 (PAI1)	GTT CTG CCC AAG TTC TCC C	TGC CAC TCT CGT TCA CCT	57 °C
CXCL8 (IL8)	GAC ATA CTC CAA ACC TTT CC	AAC TTC TCC ACA ACC CTC	60 °C
VCAM-1	GCA AGT CTA CAT ATC ACC C	AAT CTT CCA TCC TCA TAG CA	57 °C
SELE	TTG CCC TAT GCT ACA CAG	TTG AGT CCA CTG AAG CCA	56 °C
ICAM-1	GGA GCC CGC TGA GGT CAC GA	CGC TGG CAG GAC AAA GGT CTG G	66 °C
MCP-1	ATG AAA GTC TCT GCC GCC	CTT CTT TGG GAC ACT TGC T	57 °C
HO1	CAG TGC CAC CAA GTT CAA GC	GTT GAG CAG GAA CGC AGT CTT	63 °C
NQO1	CGT CCT TCA ACT ATG CCA	TTT ACC TGT GAT GTC CTT TCT G	57 °C
NOX4	CCA GTC ACC ATC ATT TCG G	AAT CGT TCT GTC CAG TCT CCT	57 °C
GAPDH	CGG GGC TCT CCA GAA CAT CAT CC	CCA GCC CCA GCG TCA AAG GTG	66 °C
TBP	GAG CCA AGA GTG AAG AAC AGT C	GCT CCC CAC CAT ATT CTG AAT CT	60 °C

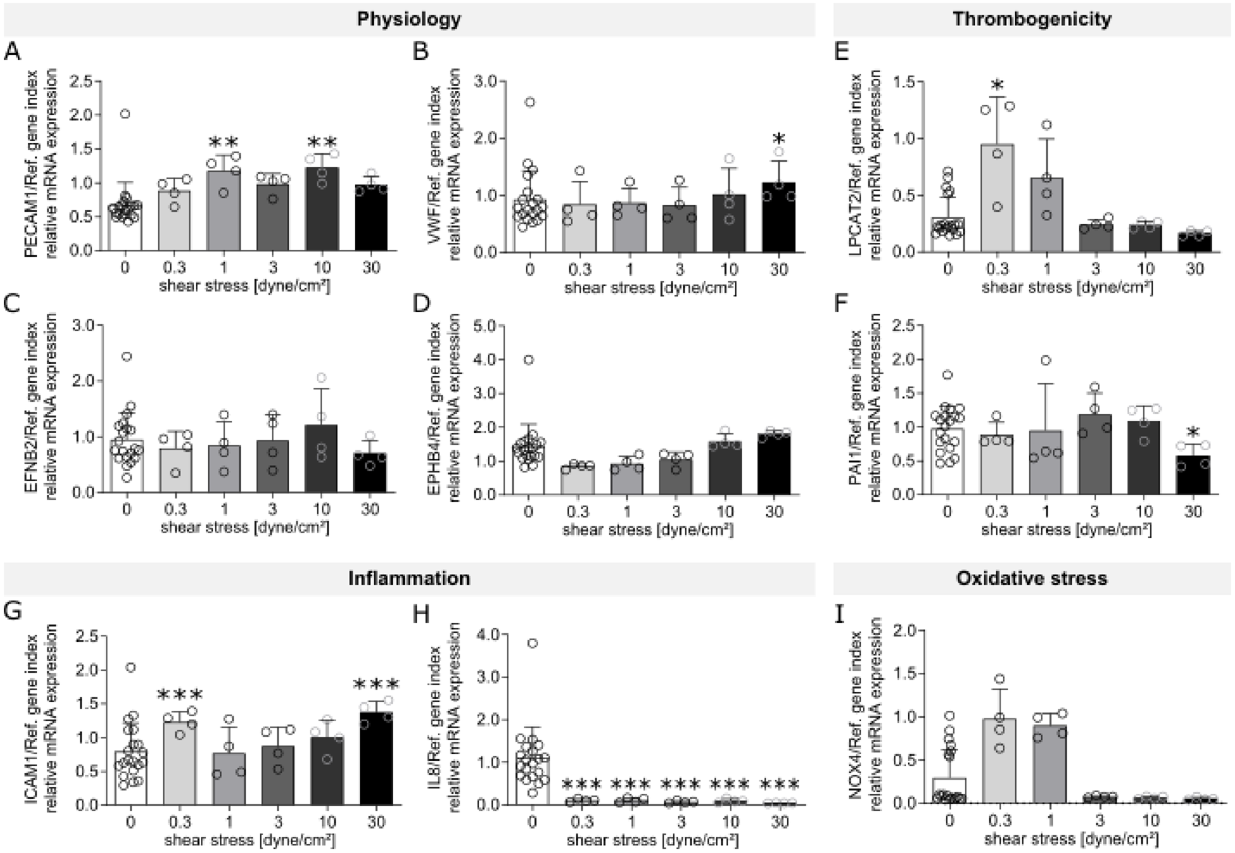

Supplementary figure S1. HUVEC mRNA expression of markers related to EC physiology: PECAM1 (A), vWF (B), EFNB2 (C), EPHB4 (D); thrombogenicity: LPCAT2 (E) and PAI1 (F); inflammation: ICAM1 (G) and IL8 (H); and oxidative stress: NOX4 (I). Statistically significant differences to the static control are indicated by asterisks (* $p < 0.05$, ** $p < 0.01$ and *** $p < 0.001$).

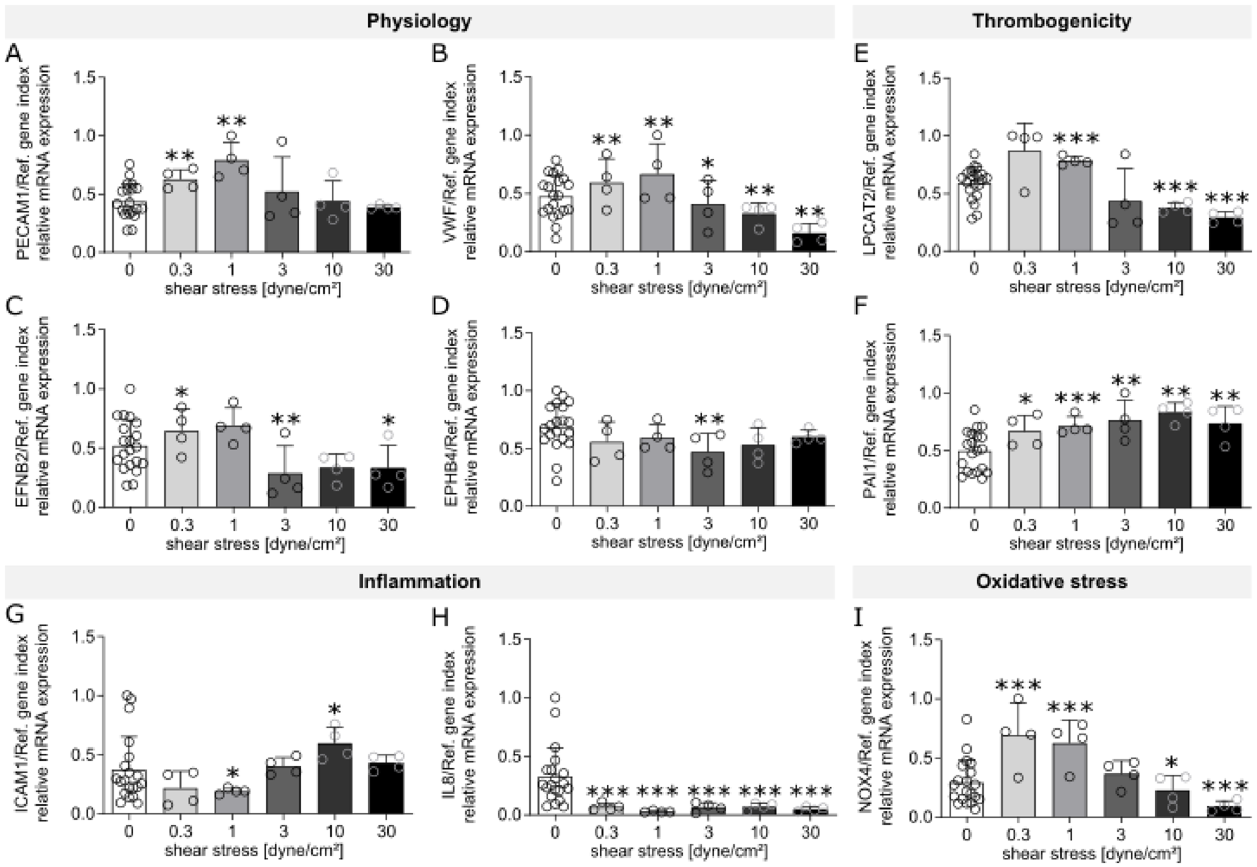

Supplementary figure S2. HUAEC mRNA expression of markers related to EC physiology: PECAM1 (A), vWF (B), EFNB2 (C), EPHB4 (D); thrombogenicity: LPCAT2 (E) and PAI1 (F); inflammation: ICAM1 (G) and IL8 (H); and oxidative stress: NOX4 (I). Statistically significant differences to the static control are indicated by asterisks (* $p < 0.05$, ** $p < 0.01$ and *** $p < 0.001$).

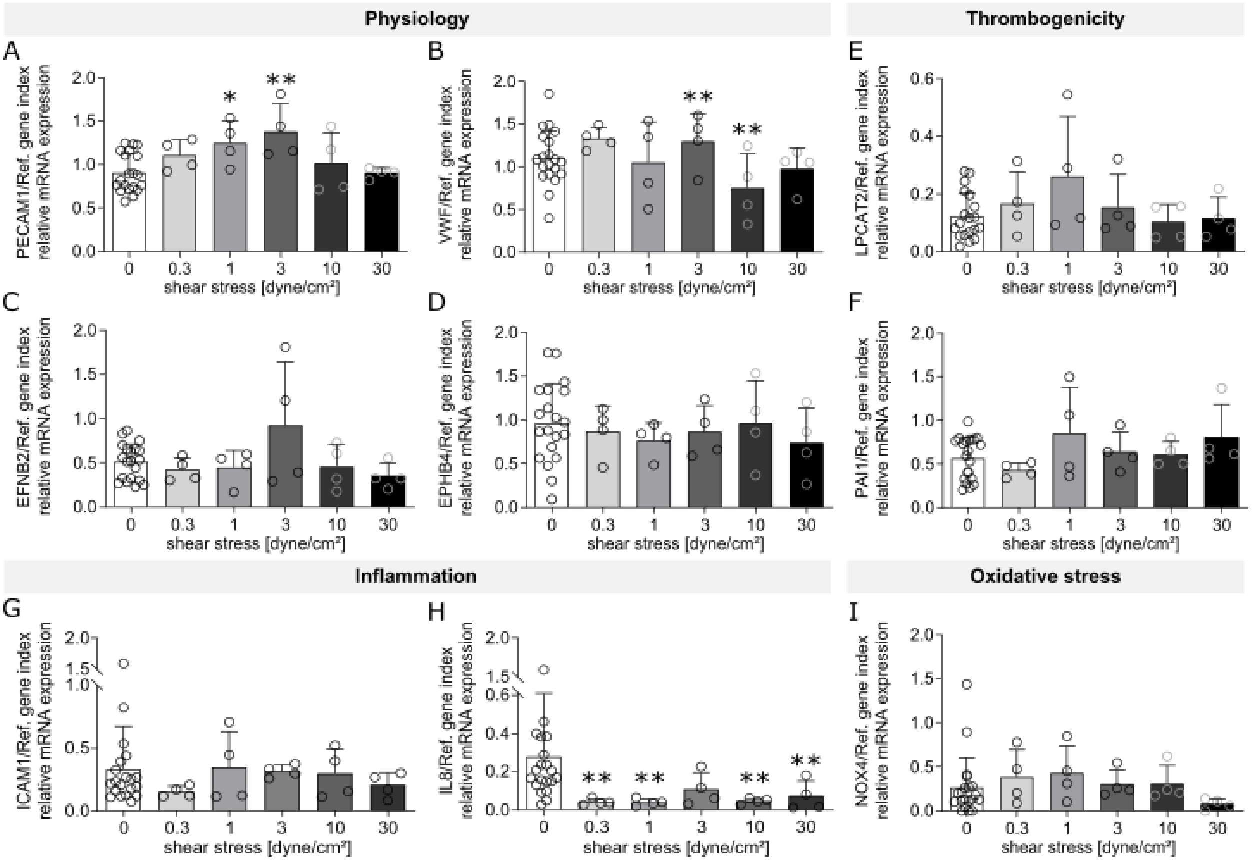

Supplementary figure S3. HAMEC mRNA expression of markers related to EC physiology: PECAM1 (A), vWF (B), EFNB2 (C), EPHB4 (D); thrombogenicity: LPCAT2 (E) and PAI1 (F); inflammation: ICAM1 (G) and IL8 (H); and oxidative stress: NOX4 (I). Statistically significant differences to the static control are indicated by asterisks (* $p < 0.05$, ** $p < 0.01$ and *** $p < 0.001$).

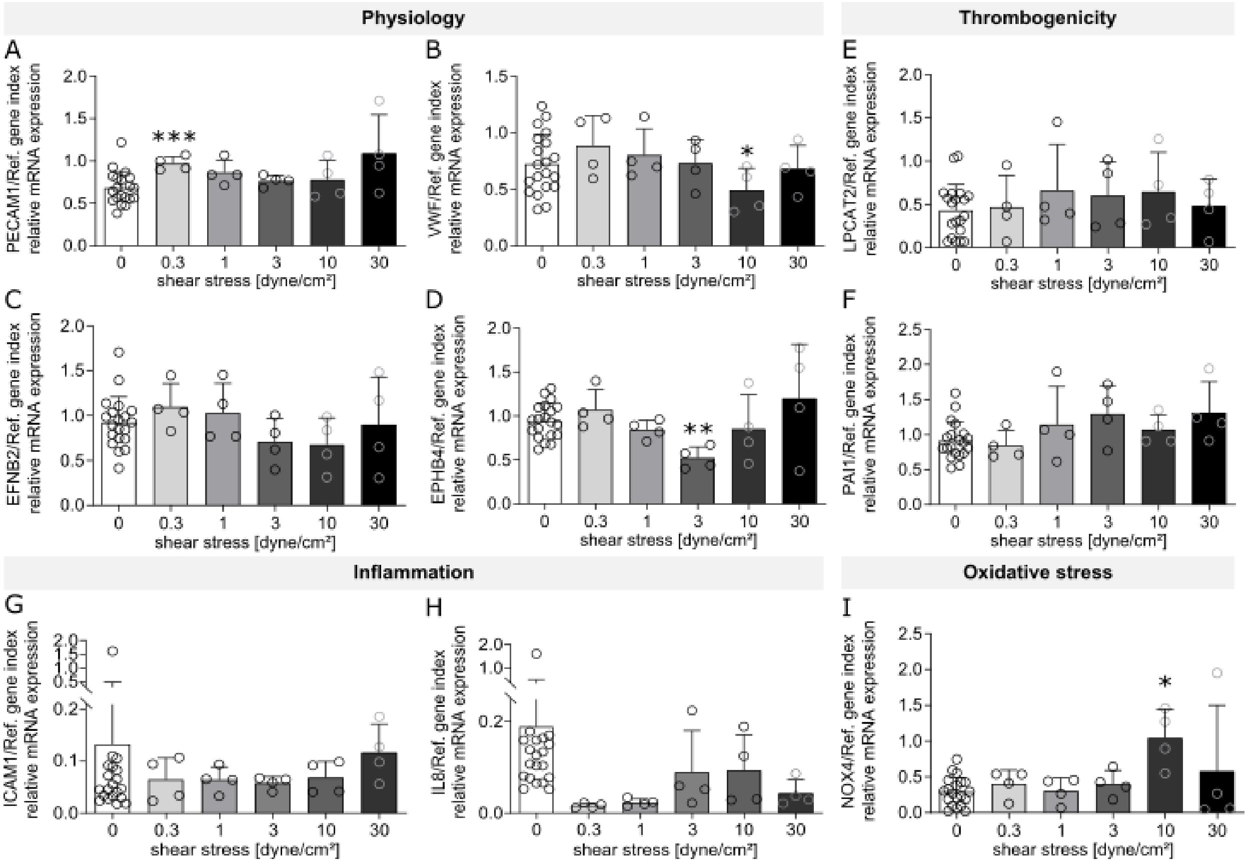

Supplementary figure S4. HPMEC mRNA expression of markers related to EC physiology: PECAM1 (A), vWF (B), EFNB2 (C), EPHB4 (D); thrombogenicity: LPCAT2 (E) and PAI1 (F); inflammation: ICAM1 (G) and IL8 (H); and oxidative stress: NOX4 (I). Statistically significant differences to the static control are indicated by asterisks (* $p < 0.05$, ** $p < 0.01$ and *** $p < 0.001$).

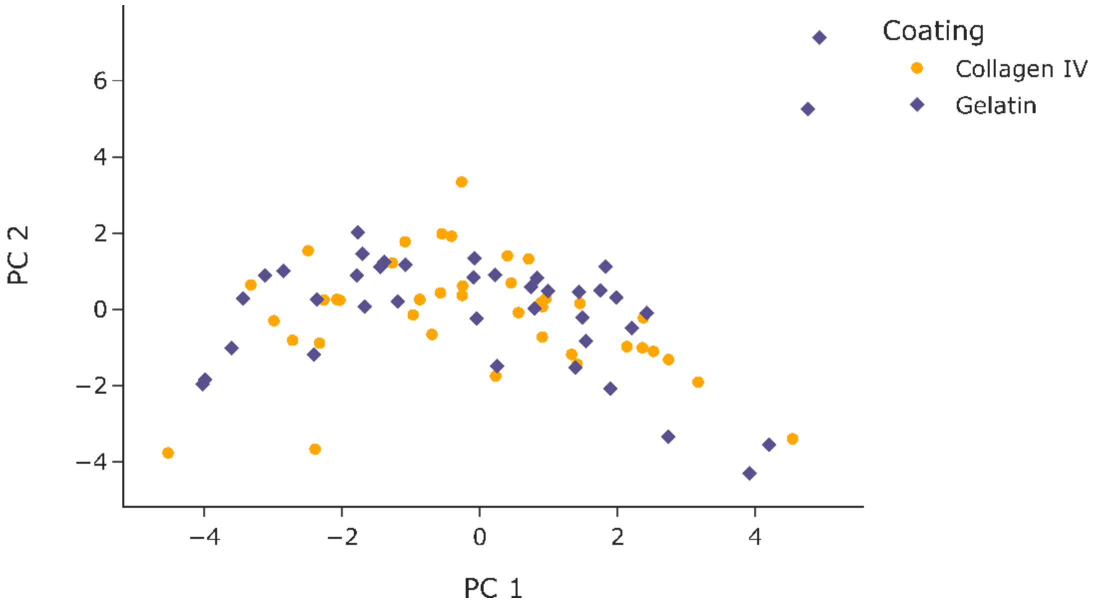

Supplementary figure S5. Principal component analysis of mRNA expression data colored for the different coatings used during cell expansion.
